# PI3K in the ventromedial hypothalamic nucleus mediates estrogenic actions on energy expenditure in female mice

**DOI:** 10.1038/srep23459

**Published:** 2016-03-18

**Authors:** Kenji Saito, Yanlin He, Yongjie Yang, Liangru Zhu, Chunmei Wang, Pingwen Xu, Antentor Othrell Hinton, Xiaofeng Yan, Jean Zhao, Makoto Fukuda, Qingchun Tong, Deborah J. Clegg, Yong Xu

**Affiliations:** 1 Children’s Nutrition Research Center, Department of Pediatrics, Baylor College of Medicine, Houston, Texas 77030, USA; 2 Department of Gastroenterology, Union Hospital, Tongji Medical College, Huazhong University of Science and Technology, Wuhan 430022, People’s Republic of China; 3 Department of Pathology, Harvard Medical School, and Department of Cancer Biology Dana-Farber Cancer Institute, Boston, MA 021151, USA; 4 Brown Foundation Institute of Molecular Medicine, University of Texas Health Science Center at Houston, Houston, TX 77030, USA; 5 Department of Biomedical Research, Diabetes and Obesity Research Institute, Cedars-Sinai Medical Center, Los Angeles, CA, USA; 6 Department of Molecular and Cellular Biology, Baylor College of Medicine, One Baylor Plaza, Houston, TX 77030, USA.

## Abstract

Estrogens act in the ventromedial hypothalamic nucleus (VMH) to regulate body weight homeostasis. However, the molecular mechanisms underlying these estrogenic effects are unknown. We show that activation of estrogen receptor-α (ERα) stimulates neural firing of VMH neurons expressing ERα, and these effects are blocked with intracellular application of a pharmacological inhibitor of the phosphatidyl inositol 3-kinase (PI3K). Further, we demonstrated that mice with genetic inhibition of PI3K activity in VMH neurons showed a sexual dimorphic obese phenotype, with only female mutants being affected. In addition, inhibition of VMH PI3K activity blocked effects of 17β-estradiol to stimulate energy expenditure, but did not affect estrogen-induced anorexia. Collectively, our results indicate that PI3K activity in VMH neurons plays a physiologically relevant role in mediating estrogenic actions on energy expenditure in females.

Estrogens play an important role in the regulation of energy homeostasis in females. The decline in circulating estrogens in post-menopausal women has been associated with development of obesity, type II diabetes and the metabolic syndrome^1^. Estrogen replacement therapy was reported to reverse the progression of obesity in post-menopausal women^2^. However, current estrogen therapy is often associated with increased risks of heart disease and breast cancer^3^. Understanding where and how estrogens act to produce anti-obesity effects may facilitate the development of highly selective therapies that can treat obesity without causing detrimental side effects.

Effects of estrogens on energy balance are primarily mediated by estrogen receptor-α (ERα)^4^,^5^. ERα knock-down in the ventromedial hypothalamic nucleus (VMH, also known as VMN) produces similar obese phenotypes seen in ovariectomized (OVX) animals^6^. Similarly, we showed that selective deletion of ERα in a subset of VMH neurons, namely steroidogenic factor-1 (SF1) neurons, leads to obesity in female mice associated with decreased energy expenditure^7^. Consistently, injections of 17β-estradiol into the VMH promote energy expenditure in female rats without altering food intake^8^. Collectively, these findings indicate that estrogens directly act on VMH neurons to regulate energy homeostasis. However, the molecular mechanisms by which VMH neurons integrate estrogen/ERα signals to regulate energy homeostasis remain to be fully elucidated.

Phosphatidyl inositol 3-kinase (PI3K) in the hypothalamus has been implicated in mediating anti-obesity effects of anorexigenic hormones, such as leptin^9^,^10^. The PI3K pathway in the hypothalamus may also mediate estrogenic actions. For example, estrogens have been shown to regulate expression of PI3K subunits in the VMH^11^. In addition, a single dose of subcutaneous injection of 17β-estradiol significantly increases phosphorylation of Akt (a downstream signal of the PI3K) in the VMH^12^. However, the physiological significance of the PI3K activity in estrogenic actions is unclear. We have generated a mouse model whose PI3K activity is selectively inhibited in VMH steroidogenic factor-1 (SF1) neurons due to the deletion of p110α, the PI3K catalytic subunit (SF1-p110α-KO)^13^. In the present study, we found that chow-fed SF1-p110α-KO males showed normal body weight balance, whereas female mutant mice developed obesity. This interesting sexual dimorphism led to the hypothesis that estrogens may be one of the hormones that drive PI3K activity in VMH SF1 neurons in females to prevent body weight gain. To test this possibility, we fully characterized energy homeostasis of female SF1-p110α-KO mice, and further examined both chronic and acute effects of 17β-estradiol in OVX SF1-p110α-KO female mice. We also used the electrophysiology approach to examine effects of estrogenic signals on cellular activity of ERα-expressing neurons in the VMH, in the absence or the presence of a PI3K blocker.

## Results

### Sexually dimorphic obesity in mice lacking PI3K activity in VMH SF1 neurons

**Figure 1:**
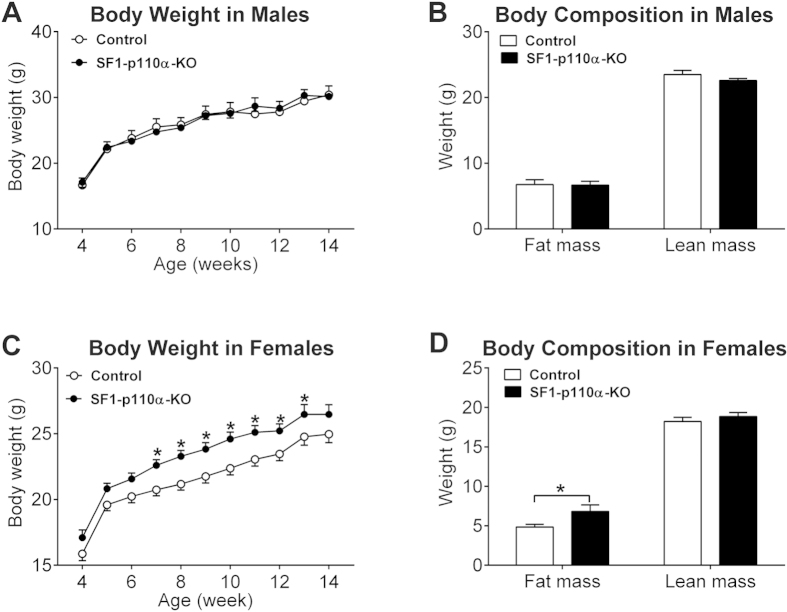
Sexually dimorphic obesity in mice lacking p110α in SF1 neurons. Body weight (**A**) and body composition (**B**) in male SF1-p110α-KO and control littermates. N = 7 or 10/genotype. Body weight (**C**) and body composition (**D**) in gonad intact female SF1-p110α-KO and control littermates. N = 38 or 44/genotype. Data are presented as mean ± SEM. *P < 0.05 between SF1-p110α-KO and control littermates in simple t-tests.

We have previously reported that male mice lacking PI3K activity selectively in VMH SF1 neurons (SF1-p110α-KO) developed obesity when fed on a high fat diet (HFD), but they showed normal body weight when fed on regular chow^13^. Consistently, we showed, in an independent study, that when fed on chow, group housed male SF1-p110α-KO mice had normal body weight compared to their control littermates (Fig. 1A). No difference in body fat mass and lean mass was observed in chow-fed male mice (Fig. 1B). In contrast, chow-fed female SF1-p110α-KO mice showed modest but significant increases in body weight compared to controls (Fig. 1C). The increased body weight in the female SF1-p110α-KO mice was primarily reflected by increased fat mass, but not lean mass (Fig. 1D).

**Figure 2:**
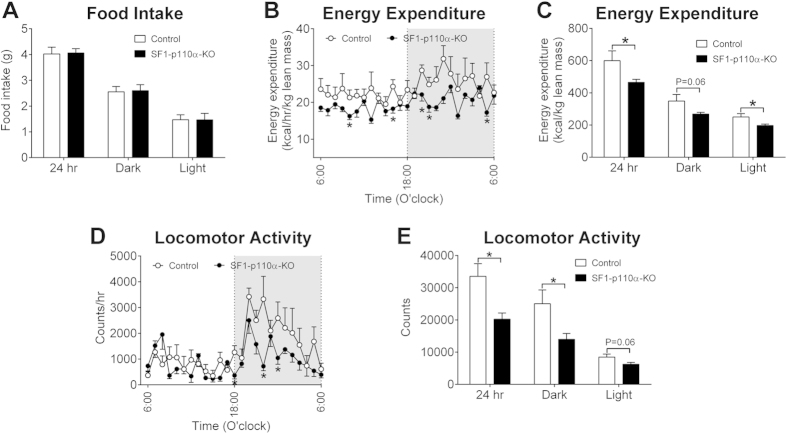
Energy homeostasis in gonad intact female mice. (**A**) Food intake during 24-hour period, the dark cycle or the light cycle in gonad intact female SF1-p110α-KO and control littermates. (**B**) Energy expenditure across the 24-hour period in gonad intact female SF1-p110α-KO and control littermates. (**C**) Cumulative energy expenditure during 24-hour period, the dark cycle or the light cycle. (**D**) Locomotor activity across the 24-hour period in gonad intact female SF1-p110α-KO and control littermates. (**E**) Cumulative locomotor activity during 24-hour period, the dark cycle or the light cycle. N = 6/genotype. Data are presented as mean ± SEM. *P < 0.05 between SF1-p110α-KO and control littermates in simple t-tests.

We then systemically characterized the energy homeostasis of chow-fed female SF1-p110α-KO mice and their controls. We found that SF1-p110α-KO females consumed comparable amount of food as controls during the 24-hour period, the dark cycle and the light cycle (Fig. 2A). On the other hand, energy expenditure of SF1-p110α-KO females was significantly lower than that of control mice at multiple time points throughout the 24-hour period (Fig. 2B). Cumulative energy expenditure during the 24-hour period and the light cycle was significantly lower in SF1-p110α-KO female mice, and cumulative energy expenditure during the dark cycle also trended to decrease in these mutant females (Fig. 2C). In addition, we detected significant decreases in locomotor activity in SF1-p110α-KO mice at multiple time points primarily during the dark cycle (Fig. 2D). Cumulative locomotor activity during the 24-hour period and the dark cycle was significantly lower in SF1-p110α-KO females than in control females, and cumulative locomotor activity during the light cycle also trended to decrease in the mutant females (Fig. 2E).

Since SF1 cells also exist in the pituitary, adrenal gland and ovary^14^, p110α may be deleted in SF1 cells in these peripheral tissues which may confound the obese phenotypes observed in the SF1-p110α-KO females. To rule out this possibility, we measured various hormones from these tissues. No significant difference was observed in serum 17β-estradiol, corticosterone, LH, FSH and T3/T4 between gonad-intact SF1-p110α-KO and control females (Table 1). Therefore, the metabolic phenotypes outlined above are not likely due to impaired PI3K signaling in the pituitary, adrenal gland and ovary, but rather to altered PI3K activity in VMH SF1 neurons.

### Effects of the ERα agonist on cellular activity in VMH ERα neurons

**Figure 3:**
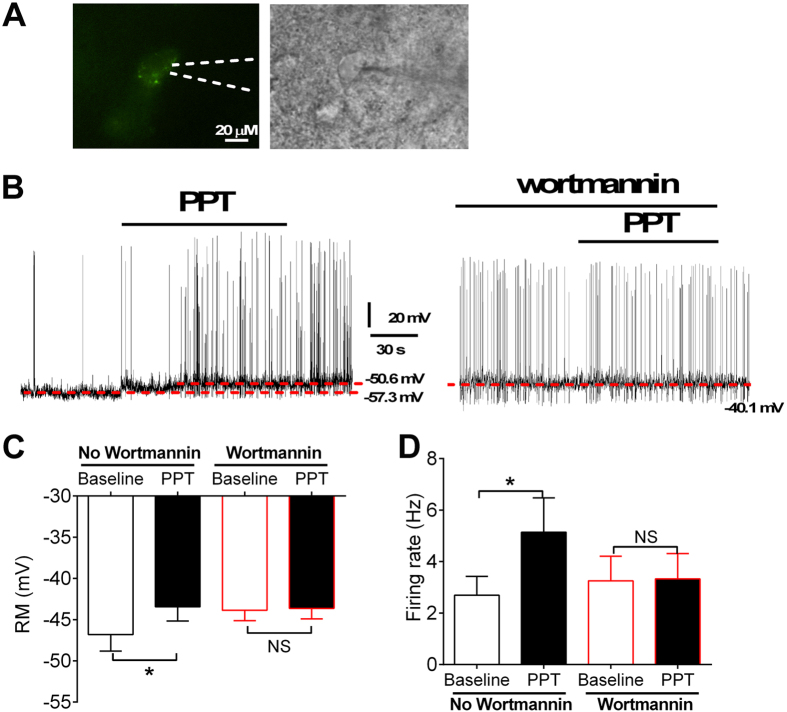
Effects of PPT on cellular activities of VMH ERα neurons. (**A**) Recording one ERα neurons in the VMH visualized under green fluorescence (left) and brightfield (right) microscopy. The scale bar = 20 μM. (**B**) Representative electrophysiological responses to PPT (100 nM bath) in the absence or the presence of wortmannin (10 μM). (**C**) Summary data for resting membrane potential. (**D**) Summary data for firing rate. N = 11 or 13/group. Data are presented as mean ± SEM. *P < 0.05 between baseline and PPT treatment in two way ANOVA analyses followed by post hoc Sidak tests.

Given the sexual dimorphism in energy balance of these SF1-p110α-KO mice and the known effects of estrogens and ERα in the VMH on energy homeostasis^6^,^7^,^8^, we speculated that estrogen may be one of the hormones that drive PI3K activity in VMH neurons in females to prevent obesity. To test this, we performed electrophysiological recordings from identified ERα-expressing neurons in the VMH using an ERα-zsGreen mouse model^15^ we recently generated (Fig. 3A). We showed that bath administration of PPT (100 nM) induced rapid depolarization in all 11 VMH ERα neurons (Fig. 3B,C). As a negative control, we found that PPT had no effects on all non-ERα neurons (ZsGreen-negative cells) in the VMH (data not shown).

To determine if PPT-induced depolarization of VMH ERα neurons requires PI3K activity, we blocked PI3K activity in these neurons with intracellular application of wortmannin (a PI3K inhibitor, 10 μM). In the presence of wortmannin, PPT failed to depolarized all VMH ERα neurons tested (n = 13; Fig. 3B,C). We also analysed effects of PPT on firing rate. We found that in the absence of wortmannin, PPT induced significant increases in firing rate in all VMH ERα neurons tested (Fig. 3B,D), while intracellular application of wortmannin blocked these PPT-induced effects (Fig. 3B,D). Together, these data indicate that the ERα agonist rapidly activates ERα-expressing neurons in the VMH, and these effects are largely mediated through PI3K activity in these neurons.

### Chronic effects of 17β-estradiol in OVX females

**Figure 4:**
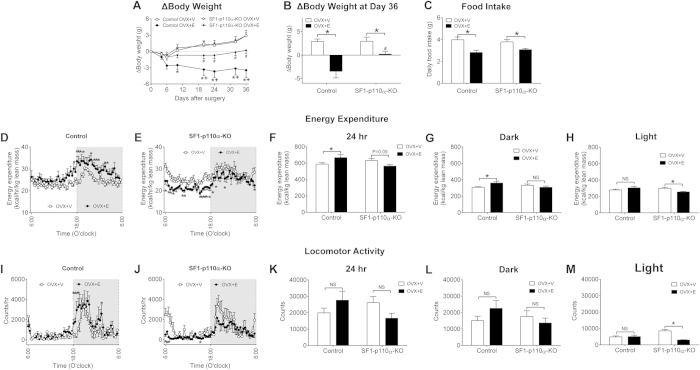
Effects of chronic 17β-estradiol supplement in OVX female mice. (**A**) Changes in body weight in OVX control and SF1-p110α-KO female mice treated with vehicle (OVX+V) or chronic 17β-estradiol supplement (OVX+E). N = 14 or 15/group. Data are presented as mean ± SEM. *P < 0.05 vs control OVX+V; ^+^P < 0.05 vs. SF1-p110α-KO OVX+E; ^#^P < 0.05 vs control OVX+V in simple t-tests. (**B**) Changes in body weight 36 days after OVX+V/OVX+E treatment. N = 14 or 15/group. Data are presented as mean ± SEM. *P < 0.05 between OVX+V and OVX+E within the same genotype; ^#^P < 0.05 between control and SF1-p110α-KO both receiving OVX+E treatment in two way ANOVA analyses followed by post hoc Sidak tests. (**D**) Averaged daily food intake in all four groups of mice. N = 9 or 10/group. Data are presented as mean ± SEM. *P < 0.05 between OVX+V and OVX+E within the same genotype in two way ANOVA analyses followed by post hoc Sidak tests. (**D**–**E**) Energy expenditure in OVX+V or OVX+E-treated control (**D**) or SF1-p110α-KO female mice (**E**). N = 5/group. Data are presented as mean ± SEM. *P < 0.05 between OVX+V and OVX+E within the same genotype in simple t-tests. (**F**–**H**) Energy expenditure during the 24-hour period (**F**), the dark cycle (**G**) or the light cycle (**H**) in all four groups. N = 5/group. Data are presented as mean ± SEM. *P < 0.05 between OVX+V and OVX+E within the same genotype in two way ANOVA analyses followed by post hoc Sidak tests. (**I,J**) Locomotor activity in OVX+V or OVX+E-treated control (**I**) or SF1-p110α-KO female mice (**J**). N = 5/group. Data are presented as mean ± SEM. *P < 0.05 between OVX+V and OVX+E within the same genotype in simple t-tests. (**K**–**M**) Locomotor activity during the 24-hour period (**K**), the dark cycle (**L**) or the light cycle (**M**) in all four groups. N = 5/group. Data are presented as mean ± SEM. *P < 0.05 between OVX+V and OVX+E within the same genotype in two way ANOVA analyses followed by post hoc Sidak tests.

To determine if PI3K in VMH SF1 neurons is required to mediate estrogenic actions on body weight balance, we characterized the metabolic phenotypes of OVX female control and SF1-p110α-KO mice that received chronic supplement of 17β-estradiol (s.c., 0.5 μg/d, OVX+E) or vehicle (OVX+V). In both control and SF1-p110α-KO mice, OVX+E treatment significantly reduced body weight compared to OVX+V mice (Fig. 4A,B). However, the body weight-lowering effects of OVX+E treatment were significantly smaller in SF1-p110α-KO mice than those in control mice (Fig. 4A,B). Further, we found that OVX+E treatment significantly reduced daily food intake compared to OVX+V treatment in both control and SF1-p110α-KO mice with similar degrees (Fig. 4C).

We then analysed the energy expenditure in all four groups. As expected, compared to OVX+V control mice, OVX+E control mice showed significantly increased energy expenditure at the multiple time points primarily during the dark cycle (Fig. 4D). Surprisingly, in SF1-p110α-KO mice, OVX+E treatment induced significant decreases in energy expenditure, primarily during the light cycle, compared to OVX+V treatment (Fig. 4E). Analyses of cumulative energy expenditure indicate that OVX+E control mice showed significantly increased energy expenditure during the 24-hour period and the dark cycle compared to OVX+V control mice, while these differences between OVX+E and OVX+V treatments were not observed in SF1-p110α-KO mice (Fig. 4F,G). On the other hand, while OVX+V and OVX+E control mice had comparable energy expenditure during the light cycle, OVX+E SF1-p110α-KO mice showed significantly decreased light-cycle energy expenditure compared to OVX+V SF1-p110α-KO mice (Fig. 4H).

We also analysed the locomotor activity in the four groups. In control mice, OVX+E treatment significantly increased locomotor activity compared to OVX+V treatment at the multiple time points primarily during the dark cycle (Fig. 4I). However, cumulative locomotor activities during the 24-hour period, the dark cycle and the light cycle were not significantly different between these two groups (Fig. 4K–M). On the other hand, in SF1-p110α-KO mice, OVX+E treatment induced significant decreases in locomotor activity at multiple time points, primarily during the light cycle, compared to OVX+V treatment (Fig. 4J). While cumulative locomotor activities during the 24-hour period and the dark cycle were not significantly different between these two groups (Fig. 4K,L), OVX+E SF1-p110α-KO mice showed significantly decreased light-cycle locomotor activity compared to OVX+V SF1-p110α-KO mice (Fig. 4M).

Collectively, these results indicate that OVX SF1-p110α-KO females were partially resistant to body weight-lowering effects of chronic 17β-estradiol supplement, associated with blunted responses to estrogenic actions to stimulate energy expenditure.

### Acute effects of 17β-estradiol in OVX females

**Figure 5:**
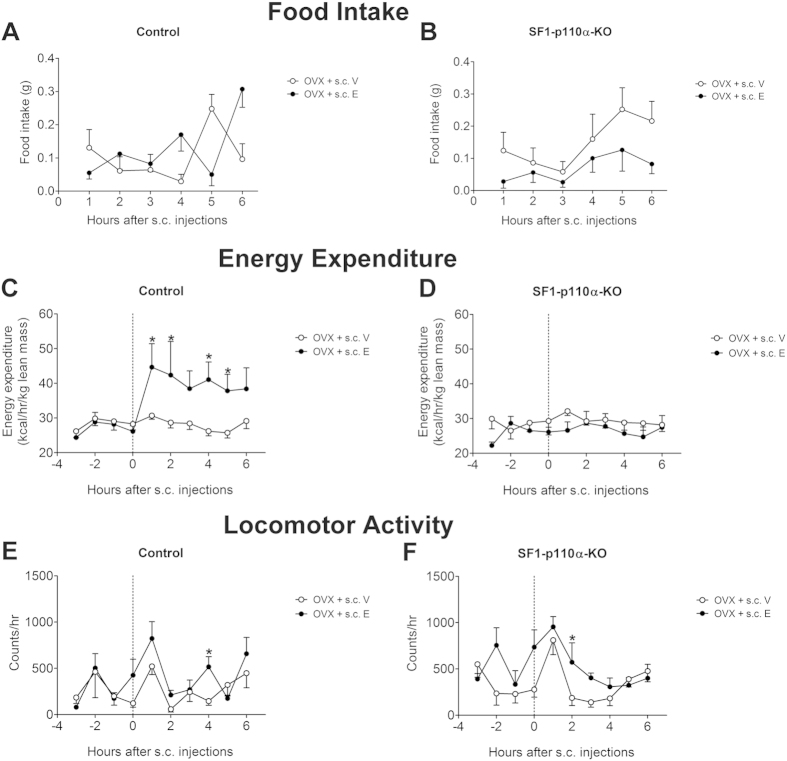
Effects of acute 17β-estradiol supplement in OVX female mice. (**A,B**) Hourly food intake in OVX control (**A**) and SF1-p110α-KO (**B**) female mice after one single dose injection of vehicle (OVX+s.c. E) or 0.5 μg 17β-estradiol (OVX+s.c. E). (**C,D**) Hourly energy expenditure in OVX control (**C**) and SF1-p110α-KO (**D**) female mice after one single dose injection of vehicle (OVX+s.c. E) or 0.5 μg 17β-estradiol (OVX+s.c. E). (**E,F**) Hourly locomotor activity in OVX control (**E**) and SF1-p110α-KO (**F**) female mice after one single dose injection of vehicle (OVX+s.c. E) or 0.5 μg 17β-estradiol (OVX+s.c. E). N = 5–7/group. Data are presented as mean ± SEM. *P < 0.05 between OVX+s.c. V and OVX+s.c. E within the same genotype at each time point in simple t-tests.

Given that the PI3K signaling and the neural firing are both rapid events, we then asked if PI3K mediates more acute effects of estrogens. To this end, we examined acute effects of a single s.c. injection of 17β-estradiol (0.5 μg) or vehicle in well-fed female OVX control mice and SF1-p110α-KO mice. Given all these mice were satiated during the light cycle, food intake in all groups was minimal and there were no significant changes in food intake (Fig. 5A,B). However, we observed a rapid and robust increase in energy expenditure in 17β-estradiol-treated control mice, effects that were not detected in SF1-p110α-KO mice (Fig. 5C,D). In parallel, 17β-estradiol induced subtle increases in locomotor activity at one time point in both control and SF1-p110α-KO mice (Fig. 5E,F). Thus, these results indicate that OVX SF1-p110α-KO females were resistant to the acute effects of 17β-estradiol to stimulate energy expenditure, while the modest effects of 17β-estradiol on locomotor activity were not affected in these mutant mice.

## Discussions

The major finding of the current study is that PI3K activity in VMH neurons is at least partly required to mediate estrogenic actions to prevent body weight gain in female mice. This notion is supported by evidence that estrogens activate VMH ERα neurons via PI3K-dependent mechanisms and more importantly that genetic inhibition of PI3K activity in VMH neurons attenuates estrogenic effects on body weight.

It has been previously reported that s.c. injections of 17β-estradiol (1 μg, 1 hour) induce pAkt in the VMH^12^, indicating that estrogens can rapidly activate the PI3K pathway in VMH neurons. Interestingly, this 17β-estradiol-induced pAkt is blocked in mice lacking ERα but restored in NERKI mice expressing a mutant ERα without the DNA binding capacity^12^. These findings not only confirm the requirement of ERα signals for the activation of the PI3K pathway, but also distinguish this rapid ERα actions from the classical ERα nuclear receptor functions which involve binding to promoters of its target genes. Taking advantage of the ERα-ZsGreen reporter mice we recently generated^15^, here we performed electrophysiological recordings from identified ERα neurons in the VMH. We showed that PPT activated all VMH ERα neurons we tested, and more importantly, these effects were largely blocked by intracellular administration of the PI3K blocker (wortmannin). These findings confirm that the estrogen-initiated PI3K activity in VMH ERα neurons is functional, at least at the level of cellular activity. In addition, these electrophysiological recordings from identified ERα neurons further support a direct action of estrogens on VMH ERα neurons. Notably, an early study recording from un-identified VMH neurons in guinea pigs reported that 17β-estradiol activates 22% VMH neurons while inhibits 38% VMH neurons^16^. Of course, the discrepancy between our observations and the early study could be attributed to species difference. Alternatively, the mixed responses from the early study could result from VMH neurons that express other ERs (e.g. ERβ). Indeed, we have recently found that the majority of ERβ-expressing neurons in the doral Raphe nuclei (DRN) are inhibited by a selective ERβ agonist^17^.

The functional relevance of PI3K in VMH neurons in estrogenic actions was further supported by observations from female SF1-p110α-KO mice. We have previously showed that deletion of p110α (the catalytic subunit of PI3K) in SF1 neurons in these mice leads to exclusive nuclear retention of FoxO1 (a downstream target of PI3K activity) in VMH neurons^13^, which therefore confirmed inhibition of PI3K activity in these neurons. Here we showed that body weight-lowering effects of 17β-estradiol supplement were significantly attenuated in OVX SF1-p110α-KO mice compared to those in OVX wild type females. These results provide genetic evidence that PI3K in VMH neurons is at least partly required to mediate effects of estrogens to reduce body weight. Notably, gonad intact SF1-p110α-KO females, with normal circulating levels of 17β-estradiol, showed modest but significant increases in body weight compared to gonad intact wild type females, while the same mutation did not affect body weight in chow-fed male mice. This sexual dimorphism further supports the notion that endogenous estrogens drive PI3K activity in VMH SF1 neurons in females to prevent obesity; therefore, lack of PI3K in VMH SF1 neurons would impair estrogenic actions on body weight regulation and lead to modest obesity in females, while the same mutation does not affect males.

It is clear that PI3K activity in VMH neurons only mediate estrogenic actions to stimulate energy expenditure, but is not involved in the regulation of food intake. The specific effects on energy expenditure were demonstrated by complete blockade of estrogenic effects to stimulate energy expenditure in SF1-p110α-KO mice, while the inhibitory effects of 17β-estradiol on food intake were not affected. The intact anorexia presumably account for remaining body weight reductions observed in OVX+E SF1-p110α-KO mice. Similarly, gonad intact SF1-p110α-KO female mice showed decreased energy expenditure but normal food intake. These findings are consistent with accumulating evidence that estrogenic actions in the VMH do not regulate food intake, but stimulate energy expenditure^6^,^7^,^8^.

The overall energy expenditure is comprised of basal metabolism, thermogenesis and activity-associated energy expenditure^18^. We suggest that PI3K in VMH neurons at least contributes to estrogenic actions on one of these components, locomotor activity. First, we found that gonad intact SF1-p110α-KO mice showed decreased locomotor activity, associated with decreased overall energy expenditure and increased body weight. Importantly, while chronic 17β-estradiol supplement stimulated locomotor activity in OVX wild type females, these effects were blocked (even reversed) in SF1-p110α-KO mice. Interestingly, both chemogenetic^19^ and optogenetic^20^ stimulation of VMH neurons promotes locomotor activity in mice. Given our observations that the ERα agonist stimulates neural activity of VMH neurons via a PI3K-dependent mechanism, we speculate that estrogen-ERα signals may activate PI3K to stimulate VMH neural activity, which in turn stimulates locomotor activity in female mice. Consistently, it was recently reported that female mice with 26% loss of ERα neurons in the VMH (due to deletion of NKX2-1) develop profound obesity, associated with decreases in locomotor activity^19^.

We also observed that a single s.c. injection of 17β-estradiol induced rapid and robust increases in energy expenditure in wild type females, and these acute responses were abolished in SF1-p110α-KO mice. Importantly, the s.c. 17β-estradiol only induced a small increase in locomotor activity in wild type females, and these effects were not affected in SF1-p110α-KO mice. The dissociation of energy expenditure and locomotor activity suggests that the robust increases in energy expenditure, at least in this acute study, were not due to locomotor activity, but rather to increased thermogenesis and/or basal metabolic rate. Thus, the blockade of these responses in SF1-p110α-KO mice suggests that PI3K activity in the VMH also mediates estrogenic actions on thermogenesis and basal metabolism. Consistently, deletion of ERα in VMH neurons resulted in impaired basal metabolism and thermogenesis^7^, while injections of 17β-estradiol into the VMH rapidly stimulate thermogenesis^8^.

It is important to note that PI3K activity in VMH neurons only partly mediates body weight-lowering effects of 17β-estradiol in female mice. Therefore, redundant mechanisms likely exist to mediate estrogenic effects on body weight. For example, other intracellular cascades, such as the JAK-STAT and AMPK pathways, have been implicated as important mediators of estrogens. Gao *et al*. demonstrated that estrogens can induce phosphorylation of STAT3 in the hypothalamus, and that selective deletion of STAT3 from the brain attenuates anti-obesity effects of estrogens in mice^21^. In particular, Martinez de Morentin *et al*. found that 17β-estradiol inhibits the AMPK pathway selectively in VMH neurons via an ERα-dependent mechanism and this inhibition mediates estrogenic actions to stimulate thermogenesis^8^. It is interesting to note that the PI3K pathway can inhibit AMPK activity through the Akt-mediated phosphorylation on the AMPK-α1 subunit^22^,^23^. Thus, it is possible that the enhanced PI3K activity may be up-stream and lead to inhibition of AMPK in VMH neurons.

The redundancy may also come from other neuronal populations. For instance, Olofsson *et al*. showed that the estrogen-induced anorexia is blunted in mice with degeneration of neuropeptide Y (NPY)/agouti-related peptide (AgRP) neurons in the arcuate nucleus^24^. Further, we showed that selective deletion of ERα in POMC neurons leads to hyperphagia in female mice, suggesting that estrogens act on ERα expressed by POMC neurons to suppress food intake^7^. Geary *et al*. showed that 17β-estradiol replacement in wild type mice suppresses food intake and potentiates CCK-induced satiation, which are accompanied by increased activity in the nucleus of solitary tract (NTS)^25^,^26^. Santollo *et al*. reported that microinjections of 17β-estradiol into the medial pre-optic area or the DRN decreases food intake in rats^27^. Finally, we recently demonstrated that deletion of ERα in the medial amygdala results in obesity associated with profound decreases in locomotor activity but normal thermogenesis and food intake^28^. Therefore, in addition to VMH neurons, actions of other neural populations certainly also contribute to the anti-obesity effects of estrogens.

In summary, we have pinpointed the rapid PI3K pathway in VMH neurons as one physiological mediator of anti-obesity effects of estrogens, and provided mechanistic insights regarding where and how estrogens act to stimulate energy expenditures without influencing food intake. These findings provide potential targets for development of novel anti-obesity therapies, at least for women.

## Methods

### Mice

SF1-Cre transgene (line 7)^29^ was bred onto Pik3ca^f/f^ mice^30^ to generate Pik3ca^f/f^/SF1-Cre (referred as SF1-p110α-KO) and Pik3ca^f/f^ littermates (controls). These mice were used for metabolic studies as outlined below. In parallel, we bred the ERα-ZsGreen mice^15^ with C57Bl6 mice to generate ERα-ZsGreen mice, which were used for electrophysiological recordings. Pik3ca^f/f^ mice were kept on a mixed C57Bl/6;129S6/SvEv background.

Mice were weaned on chow diet (6.5% fat, #2920, Harlan) at 3–4 weeks of age. All mice were housed in a 12-h light, 12-h dark cycle. Care of all animals and procedures were conformed to the Guide for Care and Use of Laboratory Animals of the US National Institutes of Health and were approved by the Animal Subjects Committee of Baylor College of Medicine.

### Body weight and body composition in male and gonad intact female mice

Body weight was measured weekly from group housed (2–4 mice per cage) male and female mice (gonad intact). Body composition was determined at 14 weeks of age using quantitative magnetic resonance (Bruker’s Minispec MQ10, Houston, TX).

### CLAMS study in gonad intact female mice

As described before^28^,^31^, food intake, locomotor activity and energy expenditure were monitored using Comprehensive Lab Animal Monitoring System (CLAMS, Columbus Instruments) in gonad intact SF1-p110α-KO and control female mice. Briefly, mice at 14 weeks of age were housed individually at room temperature (22–24 °C) under an alternating 12:12-h light-dark cycle. After adaptation for 5 days, food intake was monitored for 2 consecutive days. Physical activity was determined using a multi-dimensional infrared light beam system with beams installed on cage bottom and cage top levels. Locomotor activity was defined as breaks of any two different light beams at cage bottom level. Simultaneously, heat production was measured to determine the energy expenditure. To avoid the possible confounding effects from diverged lean mass on the energy expenditure^32^, we performed these experiments in young mice with matched lean mass and energy expenditure was normalized by lean mass as recommended^32^.

### Chronic effects of 17β-estradiol supplement

Twelve-week old female SF1-p110α-KO and control littermates were anesthetized with inhaled isoflurane. As described before^33^,^34^,^35^, these mice received bilateral OVX with subcutaneous implantations of pellets releasing vehicle (OVX+V) or releasing 17β-estradiol (0.5 μg/d for 60 days, OVX+E). Body weight and food intake was monitored daily. A portion of these mice (n = 5 per treatment X genotype) were adapted into the CLAMS chambers 5 days after the OVX surgeries. After acclimation for 5 days, energy expenditure and locomotor activity were measured for 2 consecutive days, as described above.

### Acute effects of 17β-estradiol supplement

Twelve-week old female SF1-p110α-KO and control littermates received OVX surgeries. After a 7-day recovery, these mice were adapted into the CLAMS chambers. After acclimation for 5 days, these mice received a single dose of 17β-estradiol (0.5 μg in 0.1 ml sesame oil, s.c.) or vehicle (sesame oil) at 9am. Food intake, energy expenditure and locomotor activity were monitored hourly for up to 6 hours after the injections.

### Whole-cell patch clamp

ERα-ZsGreen mice (at 6–10 weeks of age, male and female) were used for electrophysiological recordings. Briefly, mice were deeply anesthetized with isoflurane and then decapitated, and the entire brain was removed and immediately submerged in ice-cold sucrose-based cutting solution. Using a Microm HM 650V vibratome (Thermo Scientific), the brains were cut into coronal slices (without trimming at other dimension) at the thickness of 250 μm each. Usually, three to four consecutive brain slices per mouse, ranging from Bregma −2.54 mm to −1.46 mm, contained the VMH and adjacent nuclei (the ARH, the dorsal medial hypothalamus and the lateral hypothalamus) were used for recordings. The slices were recovered for 1 h at 34 °C and then maintained at room temperature in artificial cerebrospinal fluid (aCSF, adjusted to pH7.3) containing (in mM) 126 NaCl, 2.5 KCl, 2.4 CaCl_2_, 1.2 NaH_2_PO_4_, 1.2 MgCl_2_, 11.1 glucose, and 21.4 NaHCO_3_) saturated with 95% O_2_ and 5% CO_2_ before recording.

Slices were transferred to the recording chamber and allowed to equilibrate for at least 10 min before recording. The slices were superfused at 34 °C in oxygenated aCSF at a flow rate of 1.8–2 ml/min. ZsGreen (+) neurons or ZsGreen (−) neurons in the VMH were visualized using epifluorescence and IR-DIC imaging on an upright microscope (Eclipse FN-1, Nikon) equipped with a moveable stage (MP-285, Sutter Instrument). Patch pipettes with resistances of 3–5 MΩ were filled with intracellular solution (adjusted to pH 7.3) containing (in mM) 128 K gluconate, 10 KCl, 10 HEPES, 0.1 EGTA, 2 MgCl2, 0.3 Na-GTP and 3 Mg-ATP. Recordings were made using a MultiClamp 700B amplifier (Axon Instrument), sampled using Digidata 1440A and analyzed offline with pClamp 10.3 software (Axon Instrument). Series resistance was monitored during the recording, and the values were generally < 10 MΩ and were not compensated. The liquid junction potential was +12.5 mV, and was corrected after the experiment. Data were excluded if the series resistance increased dramatically during the experiment or without overshoot for action potential. Currents were amplified, filtered at 1 kHz, and digitized at 20 kHz. Frequency and peak amplitude were measured using the Mini Analysis program (Synaptosoft, Inc.). Current clamp was engaged to test resting membrane potential and firing rate at the baseline and after bath perfusion of 100 nM propyl pyrazole triol (PPT, a selective ERα agonist)^36^, with or without the intracellular application of wortmannin (10 μM), for 6 min. The concentration of PPT was chosen based on previous studies showing that effects of PPT at this dose were blocked by genetic deletion of ERα^33^. The values for resting membrane potential and firing rate were averaged within 2-min bin at the baseline or after PPT perfusion. Note that we recorded PPT effects in brain slices prepared from both male and female ERα-ZsGreen mice. Since we did not notice any differences between sexes, data from both genders were pooled for analyses.

### Serum hormones

Female mice in the estrus were anesthetized with isoflurane and rapidly decapitated in early afternoon (1 pm to 2 pm). Trunk blood was collected and centrifuged to collect serum samples. Serum samples were sent to the Hormone Assay & Analytical Services Core at Vanderbilt University for measurement of 17β-estradiol and T3/T4, to the Ligand Assay and Analysis Core at University of Virginia for LH and FSH measurement. Serum corticosterone was measured using an EIA kit (900-097, Assay Designs, Ann Arbor, MI).

### Statistics

The data are presented as mean ± SEM. Statistical analyses were performed using GraphPad Prism to evaluate normal distribution and variations within and among groups. Methods of statistical analyses were chosen based on the design of each experiment and are indicated in figure or table legends. P < 0.05 was considered to be statistically significant.

## Additional Information

**How to cite this article**: Saito, K. *et al*. PI3K in the ventromedial hypothalamic nucleus mediates estrogenic actions on energy expenditure in female mice. *Sci. Rep.* **6**, 23459; doi: 10.1038/srep23459 (2016).

## Figures and Tables

**Table 1:** Assessments of endocrine functions in gonad intact female SF1-P10α-KO and control littermates.

**Parameter**	**Control**	**SF1-p110α-KO**
17β-estradiol (pg/ml)	68.20 ± 5.63	80.50 ± 6.78
Corticosterone (ng/ml)	231.1 ± 33.1	171.1 ± 16.1
LH (ng/ml)	0.82 ± 0.11	0.81 ± 0.56
FSH (ng/ml)	21.97 ± 4.98	32.48 ± 7.17
T3 (ng/ml)	1.69 ± 0.08	1.69 ± 0.12
T4 (ng/ml)	18.64 ± 3.53	16.84 ± 1.93

All data are presented as mean ± SEM. N = 8/genotype. No statistical significance was detected between two genotypes.
